# Impact of Metal and Metal Oxide Nanoparticles on Plant: A Critical Review

**DOI:** 10.3389/fchem.2017.00078

**Published:** 2017-10-12

**Authors:** Anshu Rastogi, Marek Zivcak, Oksana Sytar, Hazem M. Kalaji, Xiaolan He, Sonia Mbarki, Marian Brestic

**Affiliations:** ^1^Department of Meteorology, Poznan University of Life Sciences, Poznan, Poland; ^2^Department of Plant Physiology, Slovak University of Agriculture, Nitra, Slovakia; ^3^SRL “Physiological Bases of Plant Productivity,” Educational and Scientific Center “Institute of Biology and Medicine,” Taras Shevchenko National University of Kyiv, Kiev, Ukraine; ^4^SI Technology, Warsaw, Poland; ^5^Department of Plant Physiology, Faculty of Agriculture and Biology, Warsaw University of Life Science—SGGW, Warsaw, Poland; ^6^Jiangsu Academy of Agricultural Sciences, Nanjing, China; ^7^National Research Institute of Rural Engineering, Water and Forests, Aryanah, Tunisia

**Keywords:** nanoparticles, nanotoxicology, oxidative stress, industrial pollutants, silver nanoparticles (Ag-NPs)

## Abstract

An increasing need of nanotechnology in various industries may cause a huge environment dispersion of nanoparticles in coming years. A concern about nanoparticles interaction with flora and fauna is raised due to a growing load of it in the environment. In recent years, several investigators have shown impact of nanoparticles on plant growth and their accumulation in food source. This review examines the research performed in the last decade to show how metal and metal oxide nanoparticles are influencing the plant metabolism. We addressed here, the impact of nanoparticle on plant in relation to its size, concentration, and exposure methodology. Based on the available reports, we proposed oxidative burst as a general mechanism through which the toxic effects of nanoparticles are spread in plants. This review summarizes the current understanding and the future possibilities of plant-nanoparticle research.

## Introduction

Nanoparticles are classified as a material in which at least one dimension is <100 nm in diameter (Auffan et al., 2009). Nanoparticles are not new to the environment and occur naturally in the form of minerals, clays, and products of bacteria. It has been used since ancient times as a colorant for metals, but the systematic design and engineering of nanoparticles for various uses has started only in the last few decades (Maurer-Jones et al., 2013). Engineered nanoparticles are designed to have the properties which are not present in bulk samples of the same materials (Auffan et al., 2009). Engineered nanoparticles are composed of a variety of materials and occur in different sizes and shapes with a suite of synthetic surface molecules, which makes them distinct from naturally occurring materials (Radad et al., 2012; Maurer-Jones et al., 2013). Metal and metal oxides nanoparticles exhibit different physiochemical properties and are different than their native bulk compounds in several respects which includes its surface, optical, thermal, and electrical properties. Metal and metal oxide nanoparticles are manufactured by addition of reducing or oxidizing/precipitating agents during their synthesis, respectively (Sanchez-Dominguez et al., 2009). Several factors are responsible for nanoparticles reactivity with biomolecules which includes nanoparticles size, core composition, shape, surface properties, purity, stability, and method of manufacturing (Teske and Detweiler, 2015; Wang P. et al., 2016). There is a good chance that nanoparticles may retain the major characteristic of their bulk material, therefore, it is needed to consider the impact of bulk material while the study of nanoparticles interaction in environment, for example, heavy metals are toxic to plants whereas silicon as a metalloid was observed to be beneficial for plants (Yadav, 2010; Tubana et al., 2016; Helaly et al., 2017).

**Figure 1:**
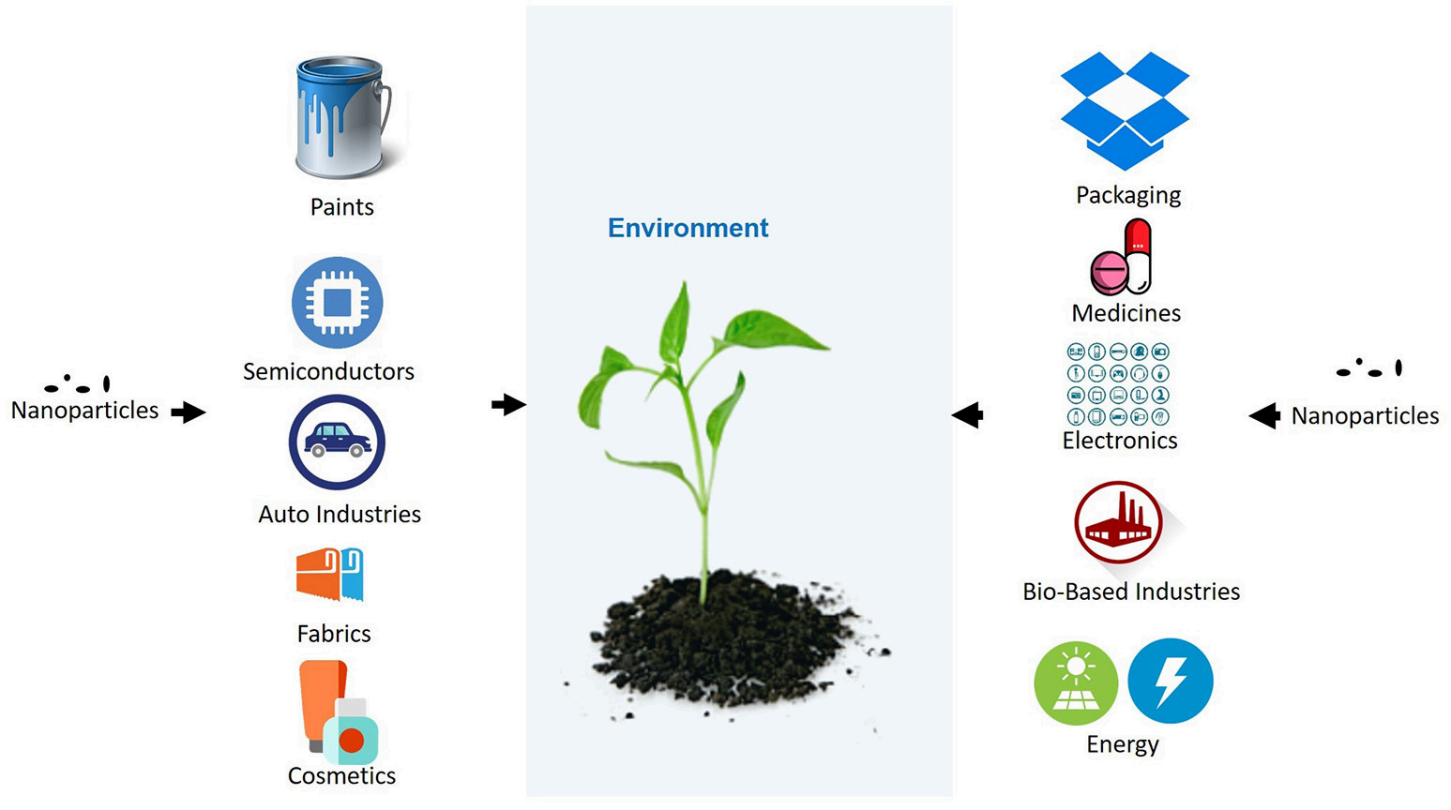
Uses of nanoparticles in different industries and its leakage to environment.

In the last decades, nanoparticles have been used in various household and industrial products. Due to the increasing use of nanoparticles in commercial products, different industries are developing novel nanoparticles for the improvement of their services and products. Some of the industries with an intensive use of nanoparticles, in which can be expected the release of nanoparticles to the environment, are indicated in Figure 1. A few of the many nanoparticles are used on a very large scale and have the potential for making its way into the environment. The nanoparticles can contaminate the environment through various processes such as, the improper management of industrial waste and improper disposal of products by the users. Several mathematical models are being developed to estimate the release of nanoparticles to the environment (Keller and Lazareva, 2014; Dumont et al., 2015). According to consumption of Silver nanoparticle (AgNP), and Zinc oxide nanoparticles in Europe per person, their release has been assumed to be significant and broadly distributed in European territory (Dumont et al., 2015). Keller and Lazareva (2014) have also estimated a significant release of different nanoparticles to the environment. In addition nanoparticles are susceptible to environmental conditions and can change their aggregation state, oxidation state, precipitation of secondary phases etc., in different environmental condition (Levard et al., 2012). The physical parameters and chemicals presence in different environment influence the stability of nanoparticles. Therefore, nanoparticles may behave differently in different condition (Levard et al., 2012), and thus their availability and reactivity in ecosystem is affected. The composition of nanoparticles may also change their properties and therefore their reactivity, penetration and translocation inside the plant which may leads to different responses of plants to the same nanoparticle, for example, Barrios et al. (2016) has shown that capping of nanoparticles influences the plant responses compared to exposure to bare nanoparticle. Plants are in continuous interaction with air, soil, and water, all of which may contain engineered nanoparticles. As the plants are also consumed by animals, the nanoparticles may be transferred to them. There is a risk that nanoparticles could invade the food chain and become dangerous to humans (the last link in the food chain). This is especially important, as the excessive usage of nanoparticles and their abundance in the environment would increase and, as a result, both plants and animals may become the source of nanoparticles for humans. Few studies in recent year confirm the trophic transfer of different nanoparticles through a terrestrial or aquatic food chain (Judy et al., 2012; Unrine et al., 2012; Hawthorne et al., 2014; De la Torre Roche et al., 2015; Tangaa et al., 2016).

Despite the plants are producers and play a major role in the ecosystem, the impact of nanoparticles upon them is not well studied (Rico et al., 2011; Feng et al., 2013; Zuverza-Mena et al., 2017). The lack of proper detection methods for nanoparticles from environments makes the study of the nanoparticles complicated (Navratilova et al., 2015; Mahdi et al., 2017). Among different possible techniques, inductively coupled plasma mass spectroscopy (ICP-MS) is one of the most reliable techniques for the detection of nanoparticles (Hadioui et al., 2015; Navratilova et al., 2015; Mahdi et al., 2017). The research performed on different plants has shown that the nanoparticles may have both a positive and a negative impact on plants, depending on size, concentration, chemical composition, zeta potential, stability, and the shape of nanoparticles (Mirzajani et al., 2013; Rafique et al., 2014; Nhan et al., 2015; Tripathi et al., 2015, 2017; Costa and Sharma, 2016; Wang Z. et al., 2016). Several studies have depicted a negative impact of nanoparticles on plants in the form of decrease in plant growth, productivity and pigments (Landa et al., 2016; Tripathi et al., 2017). On the other hand, smartly designed nanoparticles are also used for the betterment of agricultural crop production, as growth stimulators, nanopesticides, nanofertilizers, soil improving agents, or sensors for monitoring different agricultural parameters in the field (Fraceto et al., 2016; Wang P. et al., 2016). Due to the increased interest in the area, most of the research depicting the influence of industrial nanoparticles on plants has been performed in recent years. Therefore, the purpose of this review is to systematically present and analyze the research performed in the last 10 years to give an overview of the recent advancement in the field.

In the following sections, we will discuss the presence of different types of nanoparticles in the environment, the impact of different nanoparticles on plants, and a concise discussion and a general mechanism through which nanoparticles may cause an impact on the plant.

## Effect of nanoparticles on plants

Nanoparticles cover a heterogeneous range of materials (Santos et al., 2015), but only a few of them are extensively used and at present, the environment is at risk to be exposed to them. Metal and metal oxide nanoparticles of titanium dioxide (TiO_2_), silver, zinc oxide, cerium dioxide, copper, copper oxide, aluminum, nickel, and iron are most commonly used in industries and therefore are mostly studied for their impacts on different plants. Some non-metal nanoparticles, such as, single-walled carbon nanotubes and fullerene have been well studied to reveal their nanotoxicity mechanisms (Joner et al., 2008). From another side, enhancing growth together with an acceleration of seed germination for different organs of corn, tomato, rice, and soybean has been observed under exposure to single-walled carbon nanohorns (SWCNHs) (Lahiani et al., 2015). In recent years, nanoparticles have been developed to be used in agriculture as nanopesticides and nanofertilizers (which include the use of nanoparticles as nanocarrier for pesticides, fertilizers; Fraceto et al., 2016; Wang P. et al., 2016). Nanoparticles of chitosan were used to encapsulated herbicide, due to which the efficiency of herbicide was observed to be enhanced significantly (Maruyama et al., 2016). Mesoporous silicon nanoparticles as a metalloid nanoparticles have also been used to deliver DNA, proteins, and other chemicals in plants (Torney et al., 2007; Martin-Ortigosa et al., 2014). Use in agriculture includes nanoparticles, such as, nanozeolites (basic building blocks of silicate [SiO_4_]^−^ and aluminates [AlO_4_]^−^ tetrahedrons) as well as the hydrogels (consisting of different polymers such as, chitosan and alginate), which helps in the improvement of soil quality, and nanosensors (for monitoring plant and soil health; Fraceto et al., 2016). Silica nanoparticles were observed to be nontoxic to plant (Slomberg and Schoenfisch, 2012), but some authors observed the toxic effect due to decrees in pH of the media after addition of nanoparticles. Tripathi et al. (2015) have studied that Silica nanoparticle was able to alleviate chromium (VI) phytotoxicity in *Pisum sativum* (L.) seedlings (Tripathi et al., 2015). Several studies on the impact of metal and metal oxide nanoparticles on plant have shown a toxic impact on plants, whereas few studies also indicated their beneficial role in the form of enhancing plant growth parameters and productivity (Castiglione et al., 2011; Clément et al., 2013; Dimkpa et al., 2013; Jaberzadeh et al., 2013; Jiang et al., 2014; Rafique et al., 2014; Raliya et al., 2015; Okupnik and Pflugmacher, 2016; Cvjetko et al., 2017; Tripathi et al., 2017).

To determine whether the metal and metal oxide nanoparticles represent a risk to plant organisms and the environment, analytical selection of information is needed regarding size, concentration, zeta potential, uptake by a certain type of plants and effects on the plant. In particular, the zeta potential represents an important reliable indicator of nanoparticle coagulation and reactivity in solution. Zeta potential indicates total electric potential of all particles and ions in solution, and thus get effected by changes in pH or ionic strength (Teske and Detweiler, 2015). The effect of nanoparticles on plants occurs in several physiological, morphological, and genotoxic changes. Therefore, for the effective use of nanotechnology in agriculture, it is important to know the role of certain nanoparticle (Nair, 2016). Effect of different metal and metal oxide nanoparticles on different plants is observed to be variable and ranges from their positive impact to the lethal impact in plants (Tables 1–**4**). To clearly compare the different studies, it is divided into four following sections.

**Table 1:** Impact of AgNP on plants.

**Size (diameter in nm)**	**Concentration**	**Exposure methodology**	**Plant studied**	**Impact**	**References**
25	50, 500, 1,000 mg/L (phytotoxic study was performed with 1,000 mg/L)	Hydroponic, (treatment on germinated seeds)	*Oryza sativa*	- Nanoparticles broke the cell wall and damaged the vacuoles of root cells.	Mazumdar and Ahmed, 2011
20	40 gha^−1^	Field, through irrigation water, (nanoparticle applied with 10 mT magnetic field)	*Zea mays*	- Combination of silver nanoparticles and magnetic field led to improved quantitative yields of fodder maize	Berahmand et al., 2012
60	12.5, 25, 50, 100 mg/L	Hydroponic, (treatment on germinated seeds)	*Vicia faba*	- Genotoxic effect, as AgNPs exposure significantly increased the number of chromosomal aberrations, micronuclei, and decreased the mitotic index in exposed groups compared to control.	Patlolla et al., 2012
<100	250, 750 mg/L	Hydroponic, (treatment on germinated seeds)	*Cucurbita pepo*	- Reduction in plant biomass and transpiration. - Significantly reduced the pH.	Hawthorne et al., 2012
20 (polyvinylpyrrolidine-coated, PVP-NP) 6 (gum arabic coated, GA-NP)	1, 10, 40 mg/L (toxic study performed with 40 mg/L in pure culture experiment)	Petri plates (treatment on seeds)	Eleven species of common wetland plants	- PVP-NP significantly increases leave length in *Scirpus cyperinus* and *Carex lurida* whereas, decreases in *Lolium multiflorum* GA-NP shows a significant decrease in leave length except *Phytolacca americana*. - Root growth was observed to be positively affected by PVP-NP in *Phytolacca americana, Panicum virgatum*, and *Carex lurida*, whereas 6 other species has been observed to have negative effect of PVP NP. - 9 of the studied species were observed to be negatively affected by GA-NP for root growth. - PVP-NP does not have a significant impact on seed germination whereas GA-NP affect negatively for 9 studied plants whereas, *Eupatorium fistulosum* was affected positively.	Yin et al., 2012
11 ± 0.7 (Citrate)	0.05, 0.1, 1, 18.3, 36.7, 73.4 mg/L	Petri plates (treatment on seeds)	*Zea mays Brassica oleracea*	- Structural change in maize primary root cells. - Phytotoxic effect on root development. - Phytotoxic effect on root development.	Pokhrel and Dubey, 2013
18.34	0.30–60 mg/L	Growth medium with agar (treatment on germinated seeds)	*Oryza sativa*	- 60 μg/mL penetrate the cells by destroying the cell structure whereas 30 μg/mL was not able to destroy the root cells. - Up to 30 μg/mL accelerates root growth whereas 60 μg/mL restrict the root ability to grow. - Branched root systems were enhanced through the treatment of 30 μg/mL - 60 μg/mL causes decrease in chl b concentration whereas an increase in shoot carotenoid content (authors related it to antioxidant activity of carotenoids). - A decrease in total soluble carbohydrate was observed.	Mirzajani et al., 2013
10	0.2, 0.5, 3 mg/L	Growth medium with agar (treatment on seeds)	*Arabidopsis thaliana*	- Root growth inhibition. - A decrease in chlorophyll a, chlorophyll b and total chlorophyll. - Caused alteration of transcription for antioxidant and aquaporin related genes.	Qian et al., 2013
10	0.5, 1.5, 2.5, 3.5, 5 mg/kg (toxic study performed with 2.5 mg/kg)	Pots with sand (treatment on seeds)	*Triticum aestivum*	- The Ag NPs reduced the length of shoots and roots of wheat in a dose-dependent manner. Furthermore, 2.5 mg/kg of the NPs increased branching in the roots of wheat, thereby affecting plant biomass. - Accumulation of Ag was detected in the shoots, indicating an uptake and transport of the metal from the Ag NPs in the sand. - Accumulation of oxidized glutathione was observed, indicating ROS formation.	Dimkpa et al., 2013
6 and 20	0.5, 5, 10 mg/L	Hydroponic (treatment on grown plant)	*Spirodela polyrhiza*	- Dose dependent increase in levels of ROS, superoxide dismutase, peroxidase, and glutathione activity.	Jiang et al., 2014
200–800	1 mg/L	Growth medium with Agar + pots with soil (treatment on germinated seeds)	*Trigonella foenum-graecum*	- Enhancement in plant growth and diosgenin synthesis was observed.	Jasim et al., 2016
35–40	50, 75 mg/L	Pots (foliar treatment on grown plant)	*Triticum aestivum, Vigna sinensis Brassica juncea*	- Relatively unaffected (wheat) - The optimum growth promotion and increased root nodulation were observed at 50 ppm treatment (cowpea). - Improved shoot parameters were recorded at 75 ppm (brassica).	Pallavi et al., 2016
2	0, 125, 250, 500 mg/L	Petri plates (treatment on seeds)	*Raphanus sativus*	- Seed germination was not affected. - A concentration-dependent reduction in seedling elongation and water content was observed. - The seedlings exposed to 500 mg/L was observed to have significantly less Ca, Mg, B, Cu, Mn, and Zn, compared with the control. - The infrared spectroscopy analysis showed changes in the bands corresponding to lipids (3000–2800 cm^−1^), proteins (1550–1530 cm^−1^), and structural components of plant cells such as, lignin, pectin, and cellulose	Zuverza-Mena et al., 2016
20	5, 10, 20 mg/L	Hydroponic (treatment on bulb with 2–3 cm roots)	*Allium cepa*	Various chromosomal aberrations were induced in both mitotic and meiotic cells even at lower concentrations of bio-AgNPs.	Saha and Dutta Gupta, 2017
61.2 ± 33.9 (Citrate) 9.4 ± 1.3 (PVP) 5.6 ± 2.1 (CTAB)	25, 50, 75, 100 μM	Hydroponic (treatment on bulb with 2–3 cm roots)	*Allium cepa*	- Highest concentration of CTAB coted NP was observed in root, responsible for relatively higher inhibition in root growth, increase in ROS and antioxidant and DNA lysis.	Cvjetko et al., 2017
20	1000, 3000 μM	Petri plates and hydroponic (treatment on seeds)	*Pisum sativum*	- Significantly stimulated the activities of superoxide dismutase (SOD) and ascorbate peroxidase (APX) while inhibited activities of glutathione reductase (GR) and dehydroascorbate reductase (DHAR). - Declined growth parameters, photosynthetic pigments and chlorophyll fluorescence. - Nitric oxide alleviated the impact of AgNP by regulating Ag uptake, antioxidant system, oxidative stress and anatomical structures of root and shoot	Tripathi et al., 2017
12.9 ± 9.1 (90%) nanoparticles in ultrapure water	0.01, 0.05, 0.1, 0.5, 1 mg/L	Pots with soil (treatment on seedling)	*Capsicum annuum*	- Concentration dependent decrease in plant growth. - Concentration dependent increase in cytokinin concentration	Vinković et al., 2017
<100	1.5 mg/L	Hydroponic and pots (treatment on seeds)	*Triticum aestivum* (Wheat-*Pseudocercosporella herpotrichoides* Phytosystem)	- In Myronivska 808 the lipid peroxidation was observed to be significantly high where nanoparticle was present with pathogen.	Belava et al., 2017

**Table 2:** Impact of Cu and CuO NP on plants.

**Size (diameter in nm)**	**Concentration**	**Exposure methodology**	**Plant studied**	**Impact**	**References**
Around 20 (Cu nanoparticle)	200, 400, 600, 800, 1,000 mg/L	Growth medium with Agar (treatment on germinated seeds)	*Phaseolus radiates Triticum aestivum*	- Decrease in seedling and shoot growth with an increase in nanoparticle concentration. - In *P. radiates* no adverse effect on shoot growth was observed till 800mg/L concentration whereas in *T. aestivum* shoot growth was effected even at 200mg/L concentration. - Roots were more effected by the nanoparticles than the shoot.	Lee et al., 2008
30 (CuO)	0.025, 0.25, 0.5, 1, 5 mg/L	Hydroponic (treatment on plants)	*Elodea densa*	- Catalase and superoxide dismutase activities increases by 1.5 to 2 times. - stimulated photosynthesis upto 0.25mg/L level whereas suppress it above 1 mg/L concentration.	Nekrasova et al., 2011
<100 (CuO)	10, 100, 50, 1,000 mg/L	Petri plates (treatment on seeds)	Raphanus sativus Lolium perenne Lolium rigidum	- The DNA damaged was found to be increased (DNA lesions compound) with an increase in concentration of nanoparticles.	Atha et al., 2012
<50 (CuO)	0, 5, 15, 30, 45, 60, 100, 200, 400, 600, 800, 1,000, 1,500, 2,000 mg/L	Petri plates (treatment on seeds)	*Glycine max Cicer arietinum*	- A decline in root and shoot growth on above 100 mg/L concentration. - A decline in root and shoot growth on above 45 mg/L concentration.	Adhikari et al., 2012
30–40 - (CuO)	680 ± 60, 1,004 ± 120, 2,008 ± 340, 4,051 ± 950 mg/L	Hydroponic (treatment on seeds)	*Lemna gibba*	- Dose-dependent decrease in plant growth, and PS II activity. - Inactivation of PSII reaction centers, a decrease in electron transport, and an increase in thermal energy dissipation.	Perreault et al., 2014
<50 (CuO)	0.5, 1, 1.5 mM	Cotton pads shocked with growth media (treatment on seeds)	Barley	- Dose dependent reduction in shoot and root growth - Significant decrease in GSH/GSSG ratio - Increase in hydrogen peroxide and lipid peroxidation with increased concentration of NP.	Shaw et al., 2014
30 (CuO)	0.5, 1, 2, 5, 10, 20, 50, 100 mg/L	Growth media with agar (treatment on germinated seeds)	*Arabidopsis thaliana*	- Dose dependent reduction in fresh weight, root length, and total chlorophyll. - Dose dependent increase in anthocyanin content, superoxide, and hydrogen peroxide. - Loss of root gravitropism. - significant induction of genes related to oxidative stress responses, sulfur assimilation, glutathione, and proline biosynthesis	Nair and Chung, 2014
43 ± 9 (CuO)	100, 200, 500, 1,000 mg/L	Petri plates or hydroponic (treatment on seeds or germinated seeds)	*Elsholtzia splendens*	- Dose-dependent decrease in root length. - NPs were absorbed by roots and translocated to shoots. - Dose-dependent decrease in chlorophyll a, b and total chlorophyll was observed.	Shi et al., 2014
30–50 (CuO)	10 mg/L	Hydroponic (treatment on plant)	*Elodea nuttallii*	- Ultraviolet (UV) radiation treatment increases the Cu concentration in shoot. - UV radiation enhances the phytotoxic effect of nanoparticle.	Regier et al., 2015
<50 (CuO)	2.5, 10, 50, 100, 1,000 mg/L	Petri plate and hydroponic (treatment on seeds)	*Oryza sativa*	- Accumulation of nanoparticles in chloroplast. - Dose-dependent decrease in thylakoid number per grana, Photosynthetic rate, transpiration rate, stomatal conductance, maximal quantum yield of PSII photochemistry, and photosynthetic pigment contents. - Dose-dependent increase in ascorbate peroxidase and superoxide dismutase.	Costa and Sharma, 2016
40 (CuO)	10, 50, 100, 150, 200 mg/L	Hydroponic (treatment on plants)	*Lemna minor*	- Increase in peroxidase, catalase, superoxide dismutase activity. - Increase in lipid peroxidation. - Inhibition of plant growth.	Song et al., 2016
<50 (CuO)	3, 10, 30, 300 mg/Kg	Pots with sand (treatment on seeds)	Wheat	- Inhibition of root elongation by CuO NP (>10 mg/kg). - exposure resulted in root hair proliferation and shortening of the zones of division and elongation.	Adams et al., 2017
30 ± 10 (CuO)	10, 200, 1,000 mg/L	Hydroponic (treatment on plants)	Transgenic cotton (Bt-29317) Conventional cotton (Jihe321)	- Decrease in growth, development, nutrient content, indole-3-acetic acid (IAA) and abscisic acid (ABA) concentrations. - reduce the uptake of nutrients, such as, B, Mo, Mn, Mg, Zn and Fe, and inhibit the transport of Na and Mn in cotton plants. - Enhance the expression of Bt- toxin protein in leaves and roots.	Van et al., 2016
20–40 (CuO)	20, 50 mg/L	Hydroponic (treatment on seeds)	*Arabidopsis thaliana*	- Inhibit seedling growth of different ecotypes (Col-0, Bay-0, and Ws-2). - Col-0 was most sensitive ecotype to nanoparticle among three. - CuO NP was observed from root till seeds.	Wang Z. et al., 2016

### Impact of silver nanoparticles (AgNP)

Among different nanoparticles, AgNPs are fetching more attention because of their intensive uses in various products, which includes their uses as antimicrobial agents, shampoo, soap, toothpaste, waste water treatment, food packaging materials, food storage containers, fabrics, room sprays, detergents, paint, etc. (Boxall et al., 2008; Rai et al., 2009; Wijnhoven et al., 2009). Due to its extensive use, the production of nanoparticles is increasing rapidly, among which the United States itself has been reported to produce 2,500 tons/year of AgNP, of which around 150 tons end up in sewage sludge and 80 tons in surface waters (Khaydarov et al., 2009; El-Temsah and Joner, 2012). Through sludge and surface water the AgNP may easily reach to the plants.

The AgNPs of 25 nm at high concentration was observed to break the cell wall and damage the vacuoles of root cells of *Oryza sativa*, thus causing a toxic effect (Mazumdar and Ahmed, 2011). Mirzajani et al. (2013) observed that the AgNP was unable to penetrate the root cells of *O. sativa* when present in low concentration (up to 30 μg/mL), whereas the higher concentration was able to destroy the cell structure and cause the toxic effect. The authors also reported that, the 30 μg/mL accelerates root growth, whereas 60 μg/mL restrict the ability of root to grow. The observations indicate that the penetration of AgNP is necessary to cause a toxic effect, whereas when present in surrounding, it may have a positive impact on plants. Krishnaraj et al. (2012) observed a mild or no effect of biologically synthesized AgNP on *Bacopa monnieri*. The synthesized nanoparticle induced the protein and carbohydrate synthesis and decreased the total phenol contents, which can be considered as a positive effect, it may be due to the presence of different size (2–50 nm) of nanoparticle with different penetration capacity in the highest applied concentration (100 ppm), or the different chemical property of biologically synthesized NP. The AgNP of 200–800 nm size was observed to enhance the plant growth (Jasim et al., 2016), whereas 35–40 nm of AgNP was observed to positively influence the root and shoot growth of different plant (Pallavi et al., 2016), which may be due to the inability of the penetration of large nanoparticles in studied low concentration as reported by Mirzajani et al. (2013). Different size of AgNP used in the various studies shows a clear correlation between the size and toxic relation of NP to the plant, the NP with lower size was always observed to have higher toxicity to the plant compared to larger NP (Yin et al., 2012; Jiang et al., 2014; Cvjetko et al., 2017).

AgNPs (of comparatively small size i.e., <30 nm) when applied in high concentration were observed to inhibit the root and shoot growth in different plant studied (Dimkpa et al., 2013; Qian et al., 2013; Tripathi et al., 2017; Vinković et al., 2017). As a response to AgNP stress an enhancement in reactive oxygen species (ROS) was observed, which also leads to the enhanced production of antioxidant enzymes and molecules as an adaptive mechanism (Dimkpa et al., 2013; Jiang et al., 2014; Cvjetko et al., 2017; Tripathi et al., 2017). The AgNPs were also observed to cause an impact on DNA and influences the gene expression in several plants (Patlolla et al., 2012; Qian et al., 2013; Cvjetko et al., 2017; Saha and Dutta Gupta, 2017). Physiological impacts of AgNP was observed in the form of a decrease in transpiration (Hawthorne et al., 2012), chlorophyll concentration (Mirzajani et al., 2013; Qian et al., 2013; Tripathi et al., 2017); and chlorophyll fluorescence (Tripathi et al., 2017). A significant alteration in different macromolecules, lipids, proteins, lignin, pectin and cellulose were observed in *Raphanus sativus* when treated with 2 nm AgNP with 500 mg/L concentration (Zuverza-Mena et al., 2016). Plant hormones such as, cytokinin and auxin were also observed to be affected by the AgNP (Yin et al., 2012; Vinković et al., 2017).

Recent studies have shown that when AgNP was combined with different treatment/compounds, it may have a different impact on plants (Berahmand et al., 2012; Belava et al., 2017; Tripathi et al., 2017). This can be explained by the influence of other phenomena/compound on AgNP. AgNP treatment in combination with magnetic field was observed to improve quantitative yields in *Zea mays* (Berahmand et al., 2012), whereas the nitric oxide was observed to alleviate the impact of AgNP by regulating Ag uptake, an antioxidant system, oxidative stress, and anatomical structure (Tripathi et al., 2017). In the wheat-pathogen phytosystem, an enhancement of lipid peroxidation was observed, when compared with NP or pathogen alone (Belava et al., 2017). Due to its fungicidal activity, AgNP have been tested against few plant-pathogenic fungi, and their impact was found to be significant in eliminating the fungi (Jo et al., 2009). But their use in agriculture is still questionable as AgNP is known to release silver ions with its age, moreover, they can affect the biomass accumulation in soil (Johansson et al., 1998; Liu and Hurt, 2010).

The study clearly indicates AgNP exhibit an impact on different aspect of plant morphology, physiology, and biochemistry, which depends on the size, properties, and concentration of the NP in use. On the basis of the indicated studies, it can be hypothesized that for exhibiting a toxic effects AgNP need to penetrate the plant tissue and interfere with different metabolic activities. For better understanding, the influence of different AgNP on a plant is summarized in Table 1.

### Impact of copper and copper oxide nanoparticles

Copper is an essential micronutrient, which is incorporated in many proteins and enzymes, therefore, playing a significant role in plant health and nutrition. Copper nanoparticles (Cu NP) are widely used in different commercial applications such as, an antimicrobial agent, catalysts, gas sensors, electronics, batteries, heat transfer fluids, etc. (Kasana et al., 2017). Due to its oxidative property, copper oxide nanoparticles (CuO NP) are assumed to have a higher toxic effect than Cu NP. CuO NP was observed to have a positive impact on *Elodea densa* (waterweed) and stimulate photosynthesis at low concentration (<0.25 mg/L), but the impact scenario completely changes with higher doses and at 1 mg/L concentration a clear suppression in photosynthesis was observed (Nekrasova et al., 2011). The root morphology was reported to be adversely affected with Cu and CuO NP, with almost complete inhibition with a high dose of NP (Lee et al., 2008; Adhikari et al., 2012; Perreault et al., 2014; Shaw et al., 2014; Song et al., 2016; Adams et al., 2017). CuO NP was observed to enhance the production of ROS in plants (Nair and Chung, 2014; Shaw et al., 2014). Different antioxidant compounds were observed to be significantly increased in plants treated with NP indicating the activation of the protective mechanism by plants (Shaw et al., 2014; Song et al., 2016). The genetic level study on *Arabidopsis thaliana* has shown that at 0.2 mg/L concentration CuO NP does not cause any impact on the expression of genes related to oxidative stress responses, sulfur assimilation, glutathione, and proline biosynthesis (ATPS, APR, CS, GCL, P5CS1, and P5GS2), whereas, the gene expression was observed to be upregulated at higher concentrations (Nair and Chung, 2014). Atha et al. (2012) have reported a significant accumulation of oxidatively modified, mutagenic DNA lesions in different plants indicating the DNA damage as a response to CuO NP treatment. CuO NP was also observed to negatively affect the photosynthetic activity by inactivating PS II reaction centers, and causing a decrease in electron transport, thylakoid number per grana, photosynthetic rate, photosynthetic pigments, transpiration rate, stomatal conductance (Perreault et al., 2014; Costa and Sharma, 2016). Phytohormones were also observed to be altered as a response to CuO NP (Nair and Chung, 2014; Van et al., 2016). When different varieties or plants were studied together, the influence of CuO NP was observed to be different in genetically diverse plants (Lee et al., 2008; Adhikari et al., 2012; Atha et al., 2012; Van et al., 2016; Wang Z. et al., 2016). A combined treatment of the plant with ultraviolet radiation and CuO NP were observed to significantly enhance the phytotoxic effect of CuO NP (Regier et al., 2015).

The study indicates that the Cu and CuO NPs are toxic to plants when present in concentrations higher than 0.2 mg/L and it influence the growth, physiology, and biochemistry of plants.

### Impact of titanium dioxide nanoparticles (TiO_2_ NP)

TiO_2_ NP belong to the most used nanoparticles, which are used in cosmetic and skin care products, antibacterial and cleaning air products, paints, and for decomposing organic matter in wastewater (Castiglione et al., 2011; Clément et al., 2013). Few studies have been performed to indicate the influence of TiO_2_ NP shows that the TiO_2_ NP may influence plants in positive and negative ways (Table 3).

**Table 3:** Impact of TiO_2_ NP on plants.

**Size (diameter in nm)**	**Concentration**	**Exposure methodology**	**Plant studied**	**Impact**	**References**
5	300 mg/L	Pots (treatment on seeds and leaves)	*Spinacia oleraces*	- More than 60% increase in plant fresh and dry weight. - The amount of Rubisco activase increased by 42%, whereas, its activity increased 2.5 times, compared to untreated samples.	Gao et al., 2008
25	300 mg/L	Hydroponic (treatment on germinated seeds)	*Zea mays*	- Leaf growth inhibition and transpiration via physical effects on root water transport	Asli and Neumann, 2009
<100	2,000, 10,000, 20,000, 40,000 mg/L	Petri plate (treatment on seeds)	*Vicia narbonensis Zea mays*	- Decrease in root elongation. - Decrease in mitotic index. - Increase in aberration index.	Castiglione et al., 2011
14 25 140	100 mg/L	Hydroponic (treatment on plant)	*Brassica napus Triticum aestivum*	- Absorbed by plants, with Brassica having higher capacity to absorbed nanoparticle. (14 nm particle was absorbed more than 25 nm) - Moderate or no effect on plant growth. - Accumulation in roots TiO_2_-NPs with a primary diameter lower than 140 nm	Larue et al., 2012
No description	100, 200, 300 mg/L	Field (treatment on plant)	*Triticum aestivum*	- Titanium dioxide nanoparticles at 0.02% increased different agronomic traits including gluten and starch content under water deficit condition.	Jaberzadeh et al., 2013
15	100 mg/L	Petri plates (treatment on seeds)	*Linum usitatissimum*	- Reduction in root biomass, and root length. - Reduction in seed germination after 24 h.	Clément et al., 2013
21	10, 100, 1,000 mg/L	Hydroponic (treatment on bulb with 2–3 cm roots)	*Allium cepa*	- Concentration dependent increase in genotoxicity.	Demir et al., 2014
90–98	12.5, 25, 50, 100 mg/L	Hydroponic, (treatment on bulb with 2–3 cm roots)	*Allium cepa*	- Concentration dependent increase in ROS. - Concentration dependent increase in genotoxicity.	Pakrashi et al., 2014
11.93–18.67	0, 20, 40, 60, 80, 100 mg/Kg	Pots with soil (treatment on seeds)	*Triticum aestivum*	- Increase in root and shoot length with the treatment of 60 mg/Kg or less. - Decrease in root and shoot length above 60 mg/Kg concentration.	Rafique et al., 2014.
25 ± 0.64	0, 100, 250, 500, 750, 1,000 mg/Kg	Pots with soil (treatment on plant)	*Solanum lycopersicum*	- Up to a 250 mg/Kg promoted the plant height, root length, and biomass. - Lycopene content and fruit yield was maximum for 100 mg/Kg. - Chlorophyll concentration increases up to 750 mg/Kg of nanoparticle.	Raliya et al., 2015
<25	0.01, 0.1, 1, 10 mg/L	Hydroponic (treatment on plant)	*Hydrilla verticillata*	- Increase in catalase and glutathione reductase activity. - 10 mg/L concentration has shown increase in hydrogen peroxide level.	Okupnik and Pflugmacher, 2016

Due to photocatalytic properties of titanium nanoparticles, most of the studies where TiO_2_ NP was used at foliar level has shown a positive impacts on plant (Table 3; Gao et al., 2008; Jaberzadeh et al., 2013; Raliya et al., 2015). Spinach was observed to have more than 60% increase in the fresh weight and dry weight under the influence of TiO_2_ NPs (Gao et al., 2008). The author also observed the increase in amount and activity of Rubisco activase in photosynthesis. The foliar treatment of TiO_2_ NP also showed a better growth of the plant, increase in fruit yield, and chlorophyll concentration in *Solanum lycopersicum* (Raliya et al., 2015). Jaberzadeh et al. (2013) reported that TiO_2_ NP counteracts the water stress in *Triticum aestivum* by improving agronomic traits.

The smaller TiO_2_ NP was also observed to be transported by roots, it was suggested that above diameter 140 nm, TiO_2_ NPs are no longer accumulated in roots. TiO_2_ NPs with a diameter above 36 nm was observed to be accumulated in wheat root parenchyma but did not reach the stele and therefore, do not translocate to the shoot (Larue et al., 2012). A plant response to hydroponics exposure to TiO_2_ NPs may differ from the response to TiO_2_ NP-contaminated soil exposure. Colloidal suspensions of nanoparticles were observed to inhibit the leaf growth and transpiration via physical effects on root-water transport system (Asli and Neumann, 2009). At high concentration, TiO_2_ NP was observed to be toxic to plants, even in soil system (Rafique et al., 2014). The phytotoxic response was found to be similar to AgNP or CuO NP, with a decrease in plant growth, mitotic index, and an increase in ROS, antioxidant activity, and genotoxicity (Table 3). The study indicates that the impact of TiO_2_ NP on different plants depends on the concentration, ways of treatments, and size of NP. The study also indicated that availability of TiO_2_ NP for a plant is different when provided through foliar treatment than through soil or water solution. The positive influence of TiO_2_ NP was correlated with the photocatalytic activity of Ti, but the mechanism behind this interaction is so far not understood.

### Some of the notable studies on the impact of other metal and metal oxide nanoparticles

Several other nanoparticles are used in different industries to improve the product/service quality. Some of these nanoparticles are used in plant study to observe their effects on plants (Table 4). Cerium dioxide nanoparticles (CeO_2_ NP) are mainly used in automotive industries and semiconductor industry, and can interfere with the cell metabolism due to its oxidative properties. CeO_2_ NP was observed to behave differently in tomato plant when coated with citric acid, in comparison to bare CeO_2_ NP (Barrios et al., 2016). Coated NP was observed to increase chlorophyll concentration, stem length, and catalase activity. The change in behavior of NP can be attributed to different chemical properties and size of the nanoparticle due to the presence of the cap. A different study using CeO_2_ in the presence of soil organic matter confirms that the surrounding and composition influences the behavior of nanoparticle for plant (Majumdar et al., 2016). Field and soil experiments with wheat and rice have shown that application of CeO_2_ NP compromised the quality of grain (Rico et al., 2013; Du et al., 2015). The hydroponic experiment performed on cotton shows that CeO_2_ NP destroys the vascular bundles in cotton with a decrease in indole-3-acetic acid and abscisic acid (Nhan et al., 2015). The authors also observed that conventional cotton was more sustainable to CeO_2_ NP stress in comparison to transgenic cotton. Nickel oxide nanoparticle (NiO NP) was observed to induce ROS, and antioxidant molecules whereas, it was observed to reduce the concentration of plant pigments (Faisal et al., 2013; Soares et al., 2016). Zinc (Zn) and Zinc oxide nanoparticles (ZnO NP) affected plant germination and had negative effects on root elongation (Lin and Xing, 2007, 2008). ZnO NP was also observed to reduce photosynthetic efficiency and antioxidant activity whereas it induces the ROS production in the wheat plant (Tripathi et al., 2017). DNA fragmentation was also observed due to ZnO NP toxicity in *Allium cepa* (Ghosh et al., 2016). Treatment of ZnO NP at 1,000 ppm concentration was observed to promote seed germination and seedling vigor, and in turn showed early establishment in soil manifested by early flowering and higher leaf chlorophyll content, but the higher concentration of ZnO NP at 2,000 ppm was observed to have negative and toxic effect on the growth and yield of peanut (Prasad et al., 2012). In turn, Stampoulis et al. (2009) did not observe any significant effects of ZnO on *Cucurbita pepo* in studied concentration. Burklew et al. (2012) observed an increase in the expression of different stress related micro RNA in tobacco plant when exposed to Aluminum oxide nanoparticle (Al_2_O_3_ NP). NiO NP was observed to induce the apoptosis and promote the release of caspase-3 proteases from mitochondria (Faisal et al., 2013). The authors also observed that nitric oxide ameliorates the toxic effect of nanoparticle. The Iron oxide nanoparticles (Fe_3_O_4_ NP) at lower concentrations were observed to have beneficiary impact on plant and improves germination (Iannone et al., 2016; Li et al., 2016), whereas cadmium oxide nanoparticles (CdO NP) at low concentration were found to increase total amino acid production without influencing photosynthetic parameters (Vecerova et al., 2016)

**Table 4:** Impact of few other important metal and metal oxide NPs on plants.

**Nanoparticle and Size (diameter in nm)**	**Concentration**	**Exposure methodology**	**Plant studied**	**Impact**	**References**
CeO_2_ (8)	500 mg/Kg	Pots with soil (treatment on seeds)	*Oryza sativa*	- Under NP influence, rice grain contain less Fe, S, prolamin, glutelin, lauric acid, valeric acid, and starch in comparison to control. - NP could compromise the quality of rice grain.	Rico et al., 2013
CeO_2_ (10 ± 3.2)	100, 500 mg/L	Hydroponic (treatment on germinated seeds)	Transgenic cotton (Bt-29317) Conventional cotton (Jihe321)	- Reduction in Zn, Mg, Fe, and P levels in xylem sap. - decrease in indole-3-acetic acid and abscisic acid in the root of conventional cotton. - Destruction of vascular bundles. - Conventional cotton was more sustainable to CeO_2_ nanoparticle stress in comparison to transgenic cotton.	Nhan et al., 2015
CeO_2_ (8)	100, 400 mg/Kg	Field (treatment on seeds)	*Triticum aestivum*	- 400 mg/Kg of NP decreased the chlorophyll content and increased catalase and superoxide dismutase activities. - Exposure to 200 mg/Kg resulted in embryos with larger vacuoles, whereas 400 mg/Kg resulted in reduced number of vacuoles. - NP exposure changed root and leaf cell microstructures by agglomerating chromatin in nuclei, delaying flowering by 1 week, and reduced the size of starch grains in endosperm. - An increase in grain protein level was observed.	Du et al., 2015
CeO_2_-citric acid coated (8+2) CeO_2_ (8)	62.5, 125, 250, 500 mg/Kg	Pots with soil (treatment on seeds)	*Solanum lycopersicum*	- Coated NP at 500 mg/kg increased CAT activity in leaves. - At 250 mg/kg, coated NP increased total chlorophyll, chl-a, and chl-b. - At 500 mg/kg, coated and bare NP increased stem length by 13 and 9%, respectively.	Barrios et al., 2016
CeO_2_ (8)	0–500 mg/Kg	Pots with soil (treatment on seeds)	*Phaseolus vulgaris*	- Natural organic matter influences the behavior of nanoparticles in the soils. - Lower soil organic matter increased leaf cover area under NP influence. - NP increased antioxidant enzyme activities in the aerial tissues.	Majumdar et al., 2016
Al (18), ZnO (20), Zn (35), Al_2_O_3_(60)	20, 200, 2,000 mg/L	Petri plates (treatment on seeds)	*Raphanus raphanistrum subsp. Sativus, Brassica napus, Lolium perenne, Lactuca sativa, Zea mays, Cucumis sativus*	- Phytotoxic effect was observed with 2000 mg/L. The inhibition occurred during the seed incubation process rather than seed soaking stage.	Lin and Xing, 2007
Al_2_O_3_ (not mentioned)	100, 500, 1,000 mg/L	Petri plates (treatment on seeds)	*Nicotiana tabacum*	- Dose-dependent decrease in the average root length, the average biomass, and the leaf count of the seedlings. - Increase in expression of miR395, miR397, miR398, and miR399, with 1% concentration of nanoparticle.	Burklew et al., 2012
NiO (23.34)	25, 50, 100, 250, 500, 1,000, 2,000 mg/L	Petri plates (treatment on seeds)	*Solanum lycopersicum*	- NiO induce apoptosis in tomato root cells. - Increase in ROS, antioxidants, and mitochondrial membrane potential. - Trigger the release of caspase-3 proteases from mitochondria.	Faisal et al., 2013
NiO (<100)	87.8, 131.7, 197.5, 296.5, 444.4, 666.7, 1,000 mg/Kg	Petri plates or pots with soil (treatment on seeds)	*Hordeum vulgare*	- Increase in lipid peroxidation, superoxide anion radicle, and cell death. - Decrease in leaf surface area, chlorophyll and carotenoids.	Soares et al., 2016
ZnO (20 ±5)	10, 20, 50, 100, 200, 1,000 mg/L	Hydroponic (treatment on germinated seeds)	*Lolium perenne*	- Dose-dependent inhibition of root elongation. - Above 20 mg/L concentration, a decrease in seedling biomass was observed.	Lin and Xing, 2008
ZnO (25)	400, 1,000, 2,000 mg/L	Pots or petri plates (Treatment on seeds and plant)	*Arachis hypogaea*	- Zn as a micronutrient can be delivered to plant through NP. - Up to 1,000 mg/L the NP promoted seed germination and growth vigor, whereas 2,000 mg/L was observed to be toxic for plant.	Prasad et al., 2012
ZnO (~85)	200, 400, 800 mg/L	Hydroponic (treatment on plants)	*Allium cepa*,	- Showed an increase in cytotoxicity in root cells. - An increase in DNA fragmentation reported. - Observation indicated an increase in ROS and glutathione peroxidase production, whereas a decrease in catalase.	Ghosh et al., 2016
ZnO (15.37)	100, 200 μM	Hydroponic (treatment on plants)	*Triticum aestivum*	- Reduced photosynthetic efficiency. - Increase in hydrogen peroxide and lipid peroxidation. - Inhibition of antioxidant activity. - Nitric oxide ameliorates the nanoparticle toxic effect.	Tripathi et al., 2017
Fe_3_O_4_ (10)	5, 10, 15, 20 mg/L	Petri plate and hydroponic (treatment on seeds)	*Triticum aestivum*	- NP exposure did not alter germination, plant growth and chlorophyll content. - Plant exposed to NP showed a favorable response to prevent oxidative damage	Iannone et al., 2016
Fe_3_O_4_ (17.7 ± 3.9)	20, 50, 100 mg/L	Hydroponic (treatment on seeds)	*Zea mays*	- Germination index was observed to be higher with 20 and 50 mg/L NP treatment whereas decreases with 100 mg/L treatment.	Li et al., 2016
CdO (7–60)	2.03 ± 0.45 × 10^5^ particles cm^−3^	Pots (treatment on plant)	*Hordeum vulgare*	- No change in total chlorophyll concentration, with minor change in Fv/Fm with (3) treatment. - Increase in total amino acids in all three cases with maximum in (3) treatment.	Vecerova et al., 2016

The study clearly indicates that the presence of industrial nanoparticles in the environment influence the plants. Despite the positive effects of some NPs, the studies clearly indicate that all kinds of nanoparticles represent the possible environmental risk.

## Mechanism of nanoparticle-plant interaction

Based on the scientific works performed, it is evident that most of the nanoparticles are toxic to the plants in high concentration. It is hypothesized that, for exhibiting the toxic effect the uptake of nanoparticles by plant and their translocation into different tissues is needed. Further, based on their transportation, properties, and reactivity, the nanoparticles may interfere with different metabolic activity to produce an impact on plants.

### Nanoparticle uptake

The nanoparticles when present in higher concentrations are observed to damage the plant cell wall and plasma membrane, thus penetrating it and interacting with the different plant's processes (Mazumdar and Ahmed, 2011; Mirzajani et al., 2013). Nanoparticles can enter plant tissue either through root or the above ground parts including root junctions and wounds. For uptake and translocation nanoparticles has to go through various chemical and physiological barriers. When nanoparticles interact with plant, cell wall is the first barrier it has to cross. Plant cell walls are a structure which is composed of cellulose which permits the entry of small particles and restricting the larger one, therefore smaller nanoparticles can go through this layer in a comparatively easy way in respect to larger nanoparticles. The size exclusion limit for the plant cell wall is between 5 and 20 nm (Dietz and Herth, 2011). Some of the nanoparticles have been reported to induce the formation of larger pores in cell wall which further facilitate the entry of large nanoparticles (Navarro et al., 2008; Kurepa et al., 2010). From the cell wall the nanoparticles may move through endocytosis (Etxeberria et al., 2006), and further, through the symplastic transport, it may travel to different plant tissues (Ma et al., 2010). Recently, Wong et al. (2016) have proposed a mathematical model which indicates lipid exchange mechanism for nanoparticle transport inside the plant cells. The study indicated that size, magnitude, and zeta potentials are key in determining the transport of nanoparticle inside the plant.

### Nanoparticle-plant interaction pathways

**Figure 2:**
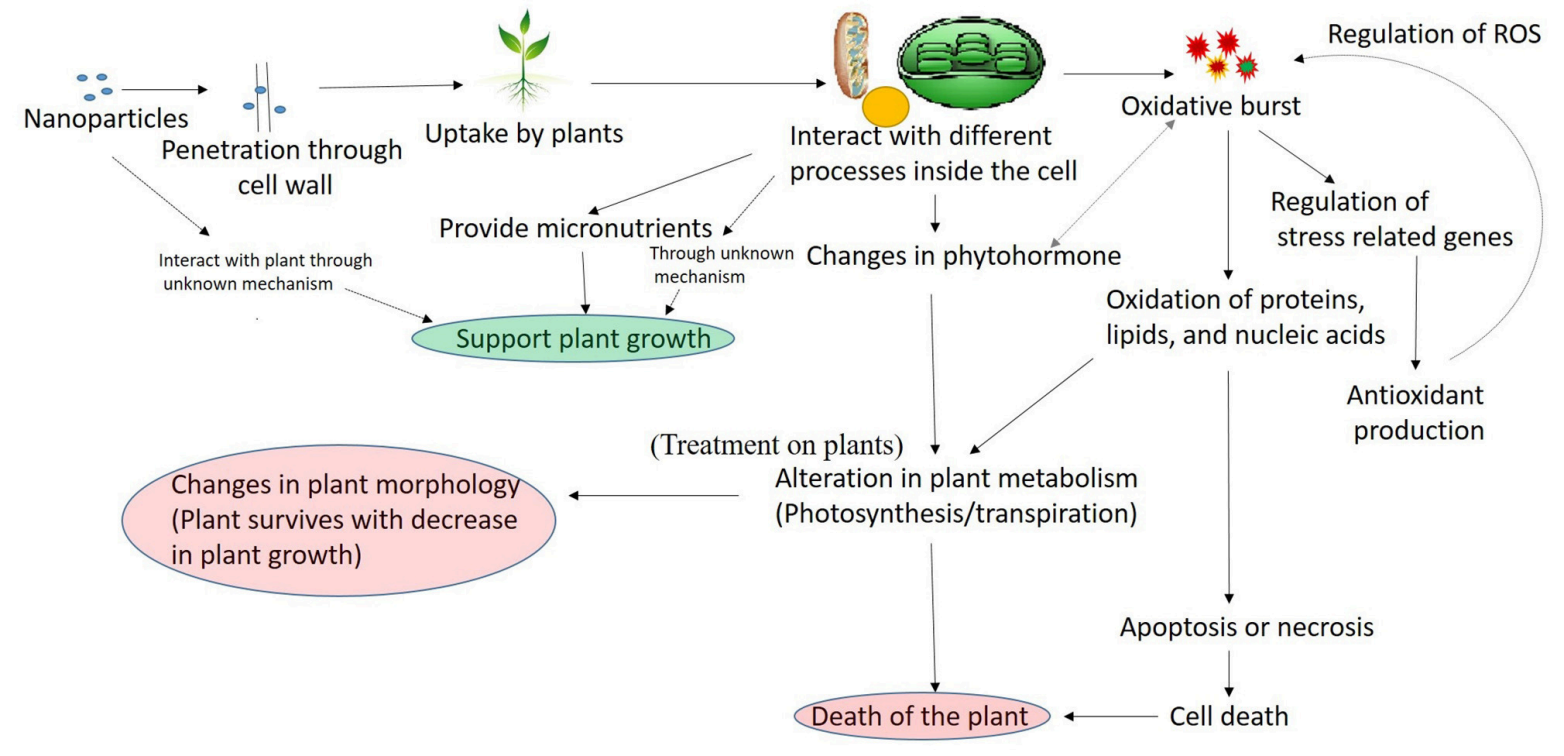
A general mechanism of nanoparticles interaction with plant.

Nanoparticles may interfere with plant metabolism in several ways, such as, by providing micronutrients (Liu and Lal, 2015), regulation of genes (Nair and Chung, 2014), or interfering with different oxidative processes in plants which results in oxidative burst (Figure 2; Hossain et al., 2015). From the previous part of this article, it is clear that several nanoparticles when present in excess results into ROS production, and interfere with the oxidative mechanism, whereas other types of interactive pathways are not deciphered and there is still much work needed to understand the other pathways. Therefore, the oxidative part is elaborated in Figure 2 and further discussed here.

The entered nanoparticles can interfere with electron transport chain of mitochondria and chloroplast, which may results into the oxidative burst, observed by the increase in ROS concentration (Dimkpa et al., 2013; Faisal et al., 2013; Jiang et al., 2014; Pakrashi et al., 2014; Cvjetko et al., 2017). It has been previously reported that under influence of different stress factors, the rate of carbon fixation is limited, which causes an increase in photoinhibition potentially steering the photosystem toward overproduction of superoxide anion radicals and H_2_O (Foyer and Noctor, 2005). Once the ROS is produced as the result of nanoparticle interaction, it is known that it interacts with almost all cellular components producing protein modifications, lipid peroxidation, and damage to DNA (Van Breusegem and Dat, 2006). Several reports have shown an increase in lipid peroxidation and DNA damage in plants-nanoparticle interaction (Atha et al., 2012; Belava et al., 2017; Cvjetko et al., 2017; Saha and Dutta Gupta, 2017), which confirms that plant interaction with nanoparticles leads into lipid peroxidation. The increased production of ROS can either induce apoptosis or necrosis (Van Breusegem and Dat, 2006; Rastogi and Pospíšil, 2012; Faisal et al., 2013), which results in plant cell death. Despite their destructive activity, ROS are also known to have a signaling role in a variety of cellular processes including tolerance to environmental stresses (Sharma et al., 2012). The destructive or signaling role of ROS depends on the equilibrium between ROS production and scavenging. Due to the multifunctional roles of ROS, the cells have developed a strong antioxidant mechanism to precisely control the level of ROS. The antioxidant mechanism contains the production of enzymatic (superoxide dismutase, catalase, and guaiacol peroxidase) and non-enzymatic (Ascorbate, glutathione, carotenoids, tocopherols, and phenolics) molecules (Sharma et al., 2012). To cope up with the stress plants increases the production of antioxidant molecules (Rastogi and Pospíšil, 2010; Sharma et al., 2012). Several reports have shown the increased production of the antioxidant molecule in the plant under the influence of nanoparticles (Faisal et al., 2013; Jiang et al., 2014; Costa and Sharma, 2016), which confirms the regulation of antioxidant system as a response to nanoparticle interaction with plant. If the antioxidant produced are unable to control the ROS, The ROS oxidized the cell macromolecules and results into the death of the cell by apoptosis or necrosis (Sharma et al., 2012), which ultimately results in the death of the plant. The recent reports have also shown that phytohormone plays an important role in plant stress response signaling (O'Brien and Benková, 2013). The hormonal control of plant development and stress adaptation is considered to be the outcome of a complex network of synergistic and antagonistic interactions between various hormones. The ROS are also linked to hormonal signaling in a complex manner and influence each other activity (Kwak et al., 2006). Different hormonal pathways are known to be upregulated or downregulated in response to different type of stresses (O'Brien and Benková, 2013). The observed increase in cytokinin level in *Capsicum annuum* as a response to AgNP stress, and a decreases in IAA and ABA in a cotton plant in response to CuO NP indicates that nanoparticle influence the hormonal balance in plants, thus affecting the plant metabolism. Therefore, it can be said that the toxic effect of nanoparticles in the plant is mainly mediated through ROS.

The electron transport chain in mitochondria and chloroplast operates in aerobic environment, and thus the excess production of ROS impact the processes (Foyer and Shigeoka, 2011). Photosynthesis is also considered to be a good measure of overall performance of plants (Kalaji et al., 2014). It is the only energy input in plants and thereby impacts all aspects of plant metabolism and physiology. Thus, the measurement of photosynthetic pigment and activity is a good measure to access the impact of stress factors. Different reports have shown that the nanoparticles influence the photosynthetic pigment concentration and its activity in plants (Qian et al., 2013; Perreault et al., 2014; Tripathi et al., 2017). A very high concentration of nanoparticles may severely affect the photosynthesis which may result in plant growth suppression or plant death. Several reports have observed significant decrease in plant growth as the result of nanoparticle exposure (For reference see Tables 1–4). Root is the primary organ for up taking nanoparticles from soil/water are adversely affected in comparison to shoot in some plants (Pokhrel and Dubey, 2013; Qian et al., 2013; Shaw et al., 2014; Tripathi et al., 2017; Vinković et al., 2017).

## Conclusion and future perspective

In modern age, nanoparticles are used intensively and becoming a part of the human life. But due to the need of present and modern life, environment cannot be neglected. It is evident from the studies that metal and metal oxides nanoparticles in excess are harmful to plants, whereas, when present in traces it can be beneficial for plants. Therefore, the increasing concentration of nanoparticles in the environment may cause a serious impact on agriculture in future. This review took out clear information from known literature to shows an influence of metal and metal oxide nanoparticle on the plant, but there are needs of research to understand the molecular mechanism of plant nanoparticle interaction. There are few research showing the beneficial role of metal and metal oxide nanoparticles in agriculture, but the mechanism at large extant are not understood, and the studies are in its primitive stage. Therefore, a lot of study is needed before bringing the nanoparticles to the field. Most of the study performed shows morphological variation caused to plants due to metal and metal oxide nanoparticles. The study also shows a clear lack of standardization for nanoparticles phytotoxic assay. Therefore, research is needed to be done in the area to understand the impact of metal and metal oxides nanoparticle on plant physiology and molecular biology.

Nanoscience is attracting lot of research funding, some of which need to be diverted for the awareness of the people about the proper disposal of nanoparticle products. The research is also needed to be performed in the area of remediation of nanoparticle from agriculture soil and wastewater.

## Author contributions

AR, MB, and MZ discussed the idea. AR, MZ, OS, MB, and XH prepared the manuscript. HK and SM read and improved the manuscript, also helped AR in preparing the tables. AR prepared the figures. All authors read and worked on the scientific language of the manuscript.

### Conflict of interest statement

The authors declare that the research was conducted in the absence of any commercial or financial relationships that could be construed as a potential conflict of interest. The reviewer, ASP, and handling Editor declared their shared affiliation.
